# Role of Ovarian Proteins Secreted by *Toxoneuron nigriceps* (Viereck) (Hymenoptera, Braconidae) in the Early Suppression of Host Immune Response

**DOI:** 10.3390/insects12010033

**Published:** 2021-01-05

**Authors:** Rosanna Salvia, Carmen Scieuzo, Annalisa Grimaldi, Paolo Fanti, Antonio Moretta, Antonio Franco, Paola Varricchio, S. Bradleigh Vinson, Patrizia Falabella

**Affiliations:** 1 Department of Sciences, University of Basilicata, Via dell’Ateneo Lucano 10, 85100 Potenza, Italy; r.salvia@unibas.it (R.S.); carmen.scieuzo@unibas.it (C.S.); paolo.fanti@unibas.it (P.F.); antonio.moretta@unibas.it (A.M.); antonio.franco@unibas.it (A.F.); 2 Spinoff XFlies s.r.l, University of Basilicata, Via dell’Ateneo Lucano 10, 85100 Potenza, Italy; 3 Department of Biotechnology and Life Science, University of Insubria, Via J.H. Dunant 3, 21100 Varese, Italy; Annalisa.Grimaldi@uninsubria.it; 4 Department of Agricultural Sciences, University of Naples “Federico II”, 80055 Portici, Italy; paolavarricchio9599@gmail.com; 5 Department of Entomology, Texas A&M University, 370 Olsen Blvd, College Station, TX 77843-2475, USA; bvinson@tamu.edu

**Keywords:** Ovarian Proteins, host-parasitoid interaction, *Heliothis virescens*, Toxoneuron nigriceps

## Abstract

**Simple Summary:**

*Toxoneuron nigriceps* is an endoparasitoid of the tobacco budworm *Heliothis virescens*. Parasitoid strategies to survive involve different regulating factors that are injected into the host body together with the egg: the venom and the calyx fluid, containing a Polydnavirus (PDV) and Ovarian Proteins (OPs). The combination of these factors increases the success of parasitism. Although many studies have been reported on venom protein components and the knowledge on PDVs is increasing, little is known on OPs. These secretions are able to interfere early with the host cellular immune response, acting specifically on host haemocytes, cells involved in immune response. Our results show that OPs induce several alterations on haemocytes, including cellular oxidative stress condition and modifications of actin cytoskeleton, so inducing both a loss of haemocyte functionality and cell death. Overall, in synergy with PDV and venom, OPs positively contribute to the evasion of the host immune response by *T. nigriceps*.

**Abstract:**

*Toxoneuron nigriceps* (Viereck) (Hymenoptera, Braconidae) is an endophagous parasitoid of the larval stages of the tobacco budworm, *Heliothis virescens* (Fabricius) (Lepidoptera, Noctuidae). During oviposition, *T. nigriceps* injects into the host body, along with the egg, the venom, the calyx fluid, which contains a Polydnavirus (*T. nigriceps* BracoVirus: *Tn*BV), and the Ovarian Proteins (OPs). Although viral gene expression in the host reaches detectable levels after a few hours, a precocious disruption of the host metabolism and immune system is observed right after parasitization. This alteration appears to be induced by female secretions including *Tn*BV venom and OPs. OPs, originating from the ovarian calyx cells, are involved in the induction of precocious symptoms in the host immune system alteration. It is known that OPs in braconid and ichneumonid wasps can interfere with the cellular immune response before Polydnavirus infects and expresses its genes in the host tissues. Here we show that *T. nigriceps* OPs induce several alterations on host haemocytes that trigger cell death. The OP injection induces an extensive oxidative stress and a disorganization of actin cytoskeleton and these alterations can explain the high-level of haemocyte mortality, the loss of haemocyte functionality, and so the reduction in encapsulation ability by the host.

## 1. Introduction

Parasitoid insects have developed in some species a great variety of adaptations in terms of physiological integration which make them similar to parasites, which constitute the most specialized forms of zoophagy [1]. Parasitoids belong to different insect orders, such as Diptera, Coleoptera, Lepidoptera, Trichoptera, and Neuroptera but they are common above all among the Hymenopterans [2]. Parasitoids develop at the expense of a single victim, the host, killing it [3]. They can be classified by different parameters, such as the number of deposited eggs per host and the parasitization mode [3]. Their host can be an egg, a larva, a pupa or, rarely, adult insects. Parasitoids are generally defined as ectoparasitoids when the juvenile stages feed on the host from the outside of the body and endoparasitoids when the development of the parasitoid takes place inside the host body [4].

During evolution, insects have developed many defense mechanisms as a result of frequent attacks by pathogens and parasites. They have an innate immunity defense, capable of recognizing and identifying a large class of foreign organisms and responding selectively and effectively to attacks [5,6,7,8]. The non-self-recognition by insects is due to the molecular interaction between the non-self-molecules and the specific receptors distributed on the host cell surface [9,10]. When the receptors recognize and bind to the proper ligands (non-self-molecules), a signal cascade is activated into the cell, promoting the immune response through the production of antimicrobial peptides (AMPs) and the melanization process [11,12].

To overcome the immune defenses of the host, the parasitoids have developed several strategies that are generally classified as active and passive [13]. The parasitic factor responsible for the active immune suppression in the host include maternal factors such as the venom and the ovarian calyx fluid, which contain the Ovarian Proteins (OPs) and in some cases Polydnavirus (PDV) or virus-like particles (VLP), and embryonic factors such as teratocytes [4,14,15,16,17].

PDVs belong to a Polydnaviridae family with unique biological traits, whose members are divided in two groups, Ichnovirus and Bracovirus, all associated with endophagous parasitoids of lepidopteran larvae [18,19,20]. Several genes expressed, even at an early stage of infection, by PDVs in different tissues of the host, including the haemocytes, have been shown to be implicated in the active suppression of the host immune response [21,22,23,24,25,26,27]. The expression of the viral gene in the host tissues does not guarantee an escape of the immune response in the early hours following oviposition. This problem is overcome by some parasitoids by injecting (together with the egg and the venom) the OPs, produced by epithelial cells of the female parasitoid reproductive traits [28,29]. The OPs have not been investigated as exhaustively as the other parasitoid maternal factors.

In this work we investigated the role of OPs of the endophagous parasitoid, the Hymenoptera Braconidae *Toxoneuron nigriceps* (Viereck) on its natural host, the noctuid moth *Heliothis virescens* (Fabricius). The parasitoid is able to lay the egg in all the larval stages of its host which, while reaching the mature larval stage, shows a slowed development, and the inability to pupate [30,31]. This alteration of the host development that allows the development of the parasitoid larva, is due to changes in the neuroendocrine equilibrium, to the redirection of the host biochemical and metabolic processes, in a condition of severe alteration of the host’s immune response [32,33,34]. This regulation of the host physiology is induced by both maternal (fluid of the calyx and venom) and embryonic (teratocytes) factors of parasitic origin [35,36]. The expression of *T. nigriceps* BracoVirus (*Tn*BV) genes into host cells determines the functional inactivation of the prothoracic glands in the mature larvae [33,34,35,36,37,38] and induces apoptosis in haemocytes [26,39]. The teratocytes, cells derived from the embryonic serosal membrane [40] contribute to the arrest of the host development [41] inducing the transformation of 20-hydroxyecdysone into inactive polar metabolites [31,32,42,43,44]. Among the factors of maternal origin, *Tn*BV and venom have been the most studied factors [15,24,26,44]. The virus infects different tissues like the fat body, the haemocytes and the prothoracic glands, in which it actively expresses its genes. Among the viral genes, *Tn*BV1 [45,46] and *Tn*BVank1 [24,26] seem to play an important role in the suppression of the immune response. In the parasitized host a decrease in the number of haemocytes has been observed as early as 2 h up to 24 h after the oviposition, with the lower number of haemocytes observed after 4 h [39]. Two hours after parasitization *H. virescens* haemolymph showed a high number of apoptotic cells and a very low level of cell proliferation [39]. Furthermore, as a result of parasitization, the remaining haemocytes showed strong morphological and structural changes, losing the ability to adhere to external objects and to encapsulate invading microorganisms [39]. The expression of the identified *Tn*BV genes, that are responsible for morphological alterations and apoptosis in haemocytes, always occurs at least after 3–4 h [24,26]. We hypothesize that the very early suppression of the host immune response after oviposition is correlated to both a passive mechanism, due to the presence of a fibrous layer on the egg surface, and an active mechanism, due to the action of the possible synergic effect of the venom and OP mixture [47,48,49]. The venom composition was elucidated by using a combined transcriptomic and proteomic approach [15] and several proteins putatively involved in the alteration of the immune system of the parasitized host identified.

Currently, nothing is known about the function of OPs of *T. nigriceps* and their role in the alterations of the immune system of the host *H. virescens*. Here we present research we did on this aspect. In particular, we believe that OPs can interfere with the cellular immune response before *Tn*BV infects and expresses its genes in the host tissues.

## 2. Materials and Methods

### 2.1. Insect Rearing

*Toxoneuron nigriceps* parasitoid was reared according to the methodology described by Vinson et al. [50]. The larvae of the host *Heliothis virescens* were reared on an artificial diet developed by Vanderzant et al. [51] (Corn Earworm Diet, Bioserve, Frenchtown, NJ, USA). The breeding temperature was maintained at 29 °C ± 1 °C, both for non-parasitized and parasitized *H. virescens* larvae. *Toxoneuron nigriceps* adults were bred at a temperature of 25 °C ± 1 °C. In both cases a photoperiod of 16 h of light and 8 h of darkness was set with a relative humidity of 70% ± 5%. For the following analysis *H. virescens* larvae parasitized at day one of the last instar were used.

### 2.2. Calyx Fluid Collection and Ovarian Protein Purification

About 80–100 adult females of *T. nigriceps*, two weeks old, previously anesthetized on ice for 10–15 min, were used for the collection of the calyx fluid, containing *T. nigriceps* BracoVirus (*Tn*BV) and Ovarian Protein (OPs). From each female, immersed in a physiological solution of 1× Phosphate-Buffered Saline (PBS), the entire reproductive apparatus was removed, pulling away the ovipositor with a pair of forceps. The isolated ovaries, explanted by one or two females, were placed in a drop of 20 μL of 1× PBS (1 or 2 equivalent females) at 4 °C and the ovarian calix were dissected to allow the flow of the calyx fluid. The collected liquid was transferred into a 1.5 mL tube (Eppendorf, Hamburg, DE, catalogue number 0030120.086) and centrifuged at 2000× *g* for 5 min at 4 °C to remove the eggs and the calyx tissues and any other debris. The supernatant, containing OPs and *Tn*BV, was further purified with 0.45 μm Millex PVDF filters (Millipore, Burlington, MA, USA, catalogue number SLHVM33RS) followed by washing 3 times with 1 mL of 1× PBS. To separate *Tn*BV a centrifugation was carried out at 30,000× *g* for 1 h at 4 °C and subsequently the supernatant containing the OPs was concentrated by ultrafiltration in a column with cut-off 3000 (Millipore, Burlington, MA, USA, catalogue number UFC900308), by centrifuging the sample at 3000× *g* until a volume of around 500 μL was obtained. The resulting filtrate was stored at −80 °C for subsequent uses (Protocol S1).

### 2.3. Collection of Haemocytes from Larvae of H. virescens

Larvae of *H. virescens* on the third day of the last instar were anesthetized in water, sterilized in 70% ethanol (*v*/*v*) and subsequently washed in sterile deionized water. The first pair of legs was cut and the hemolymph from the wound was collected and transferred to a centrifuge tube containing 1 mL of MEAD pre-cooled solution in ice [39]. The sample was centrifuged at 400× *g* for 7 min at 4 °C. The precipitate was washed twice with a MEAD-PBS solution (1:1). Finally, the haemocytes were gently resuspended in 1 mL of Grace Insect Medium (Sigma Aldrich, St. Louis, MO, USA, catalogue number, G8142) containing FBS (Gibco, Gaithersburg, MD, USA, catalogue number A4766801) 10% and antibiotic-antimycotic 1% (Gibco, Gaithersburg, MD, USA, catalogue number 15240062).

An amount of 1 × 10^6^ haemocyte cells per well were inoculated in 24-well culture plates (Corning Incorporated, New York, NY, USA, catalogue number CLS3527-100EA). OPs (deriving from 1 or 2 equivalent females) or 1× PBS (as control) collected as described above were added to the haemocytes in the culture medium and incubated at 27 °C.

### 2.4. Cells Viability

To evaluate cell viability after the treatment with OPs, and to compare the effect of parasitization and OPs alone, trypan blue staining (Sigma Aldrich, St Louis, MO, USA, catalogue number T8154) was used on haemocytes, collected from parasitized and non-parasitized larvae. OPs (obtained from 1 or 2 equivalent females) or 1× PBS (as control) were added to the haemocytes collected from non-parasitized larvae in the culture medium and incubated at 27 °C for 24 h. Trypan blue staining was added to the cell suspension at a final concentration of 0.04% and then haemocytes were counted by Neubauer’s chamber under microscopy (Eclipse 80i, Nikon, Tokyo, Japan).

### 2.5. Light Microscopy Haemocyte Observations

The haemocytes incubated with OPs (deriving from 2 equivalent females) at different time (30 min, 1 h and 2 h) or with 1× PBS (negative control) were detached from the well transferred on slides and subjected to different staining methodologies: May Grünwald GIEMSA (Sigma Aldrich, St Louis, MO, USA, catalogue number MG500 and GS500), 2,7 dichlorodihydrofluorescein acetate (H_2_DCFDA) (Thermo Fisher Scientific, Waltham, MA, USA, catalogue number D399), and tetramethylrhodamine isothiocyanate (TRITC)-conjugated phalloidin (Sigma Aldrich, St Louis, MO, USA, catalogue number P1951) dyes. For each analyzed parameter, evaluation was performed considering five random fields in three independent replicates in which cells with alteration were counted on the total number of cells.

Haemocytes were fixed with 4% paraformaldehyde for 10 min, washed with 1× PBS, and stained with May–Grünwald dye for 15 min followed by 30 min in 5% Giemsa stain. The cells were washed three times with 1× PBS and the slides were mounted with glycerol (Sigma Aldrich, St Louis, MO, USA, catalogue number G5516) for morphologic observation.

For H_2_DCFDA staining, after fixing on slides, cells were incubated in the dark with H_2_DCFDA 10 μM for 30 min at room temperature. After washing three times in 1× PBS, the slides were mounted with glycerol.

A further staining was carried out using TRITC-conjugated phalloidin staining, the haemocytes fixed on slides, were incubated with TRITC-conjugated phalloidin diluted 50 µg/mL in 1% BSA (Sigma Aldrich St Louis, MO, USA, catalogue number 05470) for 2 h at room temperature in dark conditions and then, after three washes with 1× PBS, the slides were mounted with glycerol.

For all staining methodologies, the slides were observed microscopically with Nikon Eclipse 80i equipped with a Nikon Plan Fluor 100×/0.5–1.3 Oil Iris objective and the images, five random fields obtained in three independent replicates, were recorded with a Nikon Digital Sight DS-U1 camera, and the percentage of stained/fluorescent cells was counted on the total number of cells.

### 2.6. Chromatographic Sphere Staining

Sephadex Fine G50 chromatographic beads (Cytiva, Little Chalfont, England, GB catalogue number 17004202) were stained with the Congo Red dye (Sigma Aldrich, St Louis, MO, USA, catalogue number 75768). The beads were autoclaved in 1 mL of 1× PBS at 121 °C for 20 min and centrifuged at 13,000× *g* for 5 min. The supernatant was removed and 500 μL of a 1× PBS solution containing 20 mg/mL of Congo Red was added. The beads were autoclaved again at 121 °C for 20 min, centrifuged at 13,000× *g* for 5 min and repeatedly washed with 1× PBS to remove excess of dye.

### 2.7. Encapsulation Measurement

Before testing the effect of OPs on encapsulation, we defined the minimum time needed in *H. virescens* to obtain an adequate level of encapsulation. To assess this time we performed an assay, injecting 30 beads into 5 larvae for each, at 3 different dissection times after injection (10 min, 3 h, 6 h) for 3 replicates. The level of encapsulation reached after 6 h was found to be adequate to see the encapsulation effect in subsequent experiments. All the recovered beads were observed under the microscope (Eclipse 80i, Nikon, Tokyo, Japan).

### 2.8. Injections of OPs and Chromatographic Spheres in H. virescens Larvae and Evaluation of In Vivo Encapsulation Degree

The injections were performed on larvae of *H. virescens*, on the first day of the fifth instar larva. The larvae were anesthetized in water, sterilized in 70% ethanol (Sigma Aldrich St Louis, MO, USA, catalogue number 51976) (*v*/*v*) and subsequently washed in deionized water. Then, they were dried and placed on a Petri dish. Injection of the Sephadex Fine G 50 (50–150 μm) chromatographic beads was performed in the neck membrane, using a Hamilton syringe (Sigma-Aldrich, St Louis, MO, USA, catalogue number HAM80075-1EA) having a needle with 0.13 mm internal diameter and 0.47 mm outer diameter. The chromatographic spheres were injected at different times (10 min, 1 h or 3 h) after OPs injections or 5 μL of 1× PBS. After 6 h from the injection of the spheres, the time necessary for the formation of a complete haemocyte capsule (Figure S1), the larvae were anesthetized in water, sterilized in ethanol 70% (*v*/*v*) and washed in deionized water. Subsequently they were dried and placed in a drop of 1× PBS solution. The dissection was performed by cutting the head and longitudinally cutting in the ventral position. The chromatographic spheres attached to the tissues were collected and counted. The spheres were classified according to the degree of encapsulation as: 0 = unencapsulated (no haemocytes layer); 1 = the thickness of capsule is one or more than one layer, but less than a half of the bead’s radius; 2 = the thickness of capsule is equal or more than a half of the bead’s radius. We considered encapsulated the beads that showed after 6 h case 2, and not encapsulated when the beads showed case 0 or 1.

### 2.9. Statistical Analysis of Data

Statistical analysis was performed with one-way ANOVA (analysis of variance) and Bonferroni or Tukey post-hoc tests. Statistical differences were analyzed both among all treatments and between control and treated samples at the same experimental time. For the encapsulation assay we first verified that there was no statistical difference among the experimental groups in the percent of recovered beads after the dissection and then we compared the percent of encapsulated beads on the number of recovered beads.

## 3. Results

### 3.1. Cell Viability of the Haemocytes after Treatment with Ovarian Proteins

**Figure 1:**
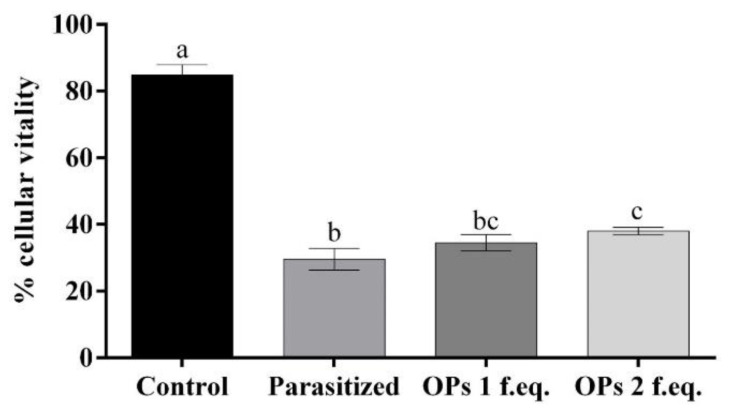
Percentage of haemocyte viability incubated with 1× PBS (control), haemocytes extracted from larvae 24 h after parasitization, haemocytes incubated with Ovarian Proteins (OPs) deriving from 1 or 2 equivalent females. Data are presented as means ± SD (*n* = 3 replicates). Statistical analysis was performed with one-way ANOVA and Tukey post-hoc test. Different letters indicate significant differences (*p* value < 0.0001).

The extracted haemocytes from non-parasitized *H. virescens* larvae (control) showed 85% of cell viability. The haemocytes extracted from larvae of *H. virescens* after 24 h from the parasitization showed only 30% of cell viability. The cell viability of haemocytes cultured in the presence of *T. nigriceps* Ovarian Proteins (OPs) for 24 h did not vary according to their concentration. The cell viability percentages equal to 34% and 38% referred to, respectively, samples treated with OPs deriving from 1 or 2 equivalent *T. nigriceps* females. Table S1 and Figure 1 show the obtained results.

### 3.2. Haemocyte Staining

#### 3.2.1. May Grunwald–Giemsa Staining

**Figure 2:**
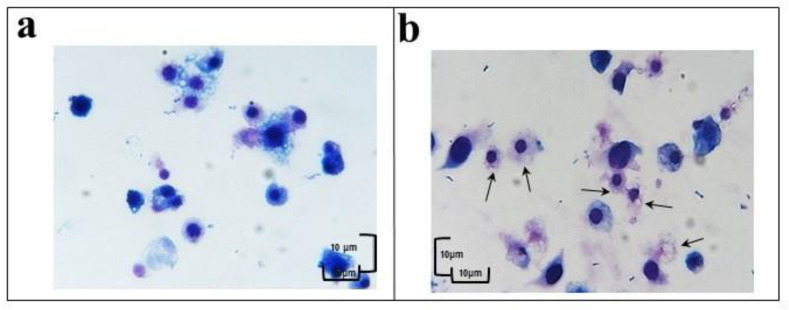
Haemocytes treated with 1× PBS (**a**) or with OPs for 2 h (**b**) stained with May Grunwald–Giemsa dye. Scale bar 10 μm. Arrows indicate vacuolization process.

**Figure 3:**
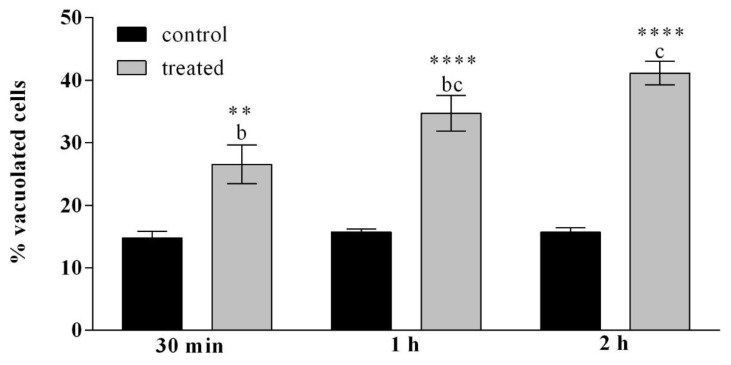
Percentage of haemocytes showing vacuolization, after treatment with OPs deriving from 2 equivalent females at different incubation times (30 min, 1 h and 2 h) and observed after May Grunwald–Giemsa (MGG) staining. Data are presented as means ± SD (*n* = 3 replicates). Statistical analysis was performed with one-way ANOVA and Bonferroni (for control vs. treatment analysis) and Tukey post-hoc test (for all sample analysis). Different letters indicate significant differences among all treatments (*p* value < 0.0001), asterisks indicate significant differences between control and treated samples at the same experimental time (** *p* value < 0.01; **** *p* value < 0.0001).

The haemocytes, treated with OPs deriving from 2 equivalent females at different incubation times (30 min, 1 h and 2 h) were observed after May Grunwald–Giemsa (MGG) staining. The cells showed a different staining as a function of the cytoplasmic pH and this allowed the different cell populations to be counted in the three different examined samples (Figure 2). The percentage of cells with acidophilic cytoplasm and massive vacuolization was greater than control and increased in relation to the different times of OP exposure (Table S2, Figure 3).

Figure 3 shows that after 30 min of incubation the percentage of cells with acidophilic cytoplasm was 27%. After 1 h the percentage was raised to 34% compared to the relative control and after 2 h the percentage reached 41%.

#### 3.2.2. H_2_DCFDA Staining

Haemocytes treated with OPs (2 equivalent females) at different times (30 min, 1 h and 2 h) were stained with 2,7 dichlorodihydrofluorescein acetate (H_2_DCFDA). H_2_DCFDA after entering into the cells, were converted into dichlorodihydrofluorescein (H_2_DCF) by intracellular esterases, H_2_DCF is rapidly oxidized to a highly fluorescent compound, 2,7-dichlorofluorescein (DCF), only in the presence of reactive oxygen species (ROS).

**Figure 4:**
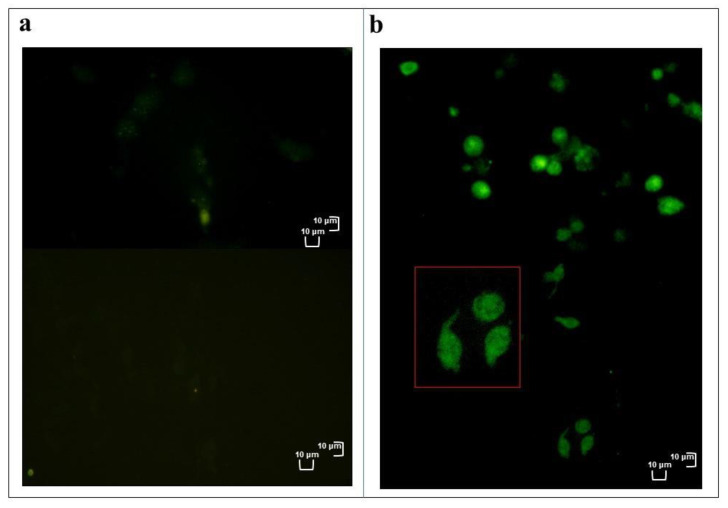
Healthy in vitro haemocytes (**a**) or treated with OPs for 2 h (**b**) and stained with 2,7 dichlorodihydrofluorescein acetate (H_2_DCFDA). Red box = enlargement of cells below. Scale bar 10 μm.

Figure 4 and Figure S2 show a strong fluorescent signal indicative of high oxidative stress in cells incubated for 2 h with OPs and a very weak fluorescent signal that could constitute the background experiment and/or could be indicative of cell physiological presence of ROS at low concentrations in control cells.

**Figure 5:**
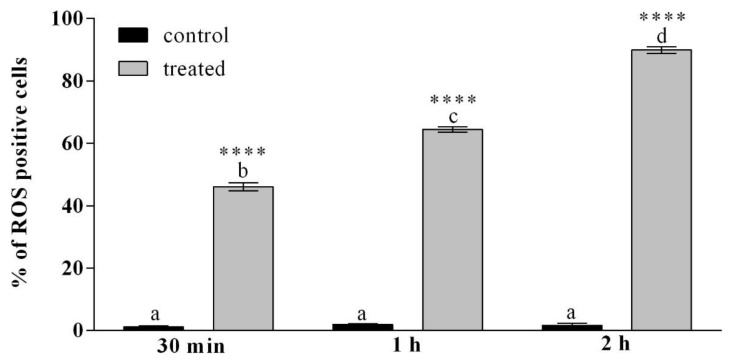
Percentage of haemocytes stained with H_2_DCFDA showing oxidative stress, after treatment with OPs deriving from 2 equivalent females at different times of exposure (30 min, 1 h and 2 h). Data are presented as mean ± SD (*n* = 3). Statistical analysis was performed with one-way ANOVA and Bonferroni (for control vs. treatment analysis) and Tukey post-hoc test (for all sample analysis). Different letters indicate significant differences among all treatments (*p* value < 0.0001), asterisks indicate significant differences between control and treated samples at the same experimental time (*p* value < 0.0001).

Table S3 and Figure 5 report the percentage of haemocytes showing a strong fluorescent signal indicative of oxidative stress, after incubation with OPs at different times. After 30 min of incubation with Ops, 46% of haemocytes were strongly fluorescent, after 1 h of treatment 64% of cells were fluorescent, after 2 h of incubation with OPs, almost the totality of the haemocytes (90%) showed oxidative stress.

#### 3.2.3. TRITC-Conjugated Phalloidin Staining

**Figure 6:**
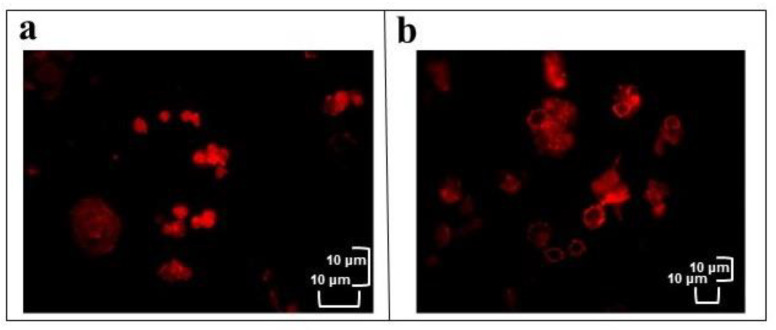
In vitro healthy haemocytes (**a**) or treated with OPs for 2 h (**b**) and stained with *TRITC-* Conjugated phalloidin. Scale bar 10 μm.

Actin filaments were detected using *TRITC-*Conjugated phalloidin staining in healthy haemocytes (control) or treated with OPs at different times (30 min, 1 h and 2 h) and stained with phalloidin. Figure 6 shows in the treated cells a weak and fragmented signal localized near the nucleus and along the plasma membrane while a strong and homogeneously distributed fluorescent signal is observed in control cells.

**Figure 7:**
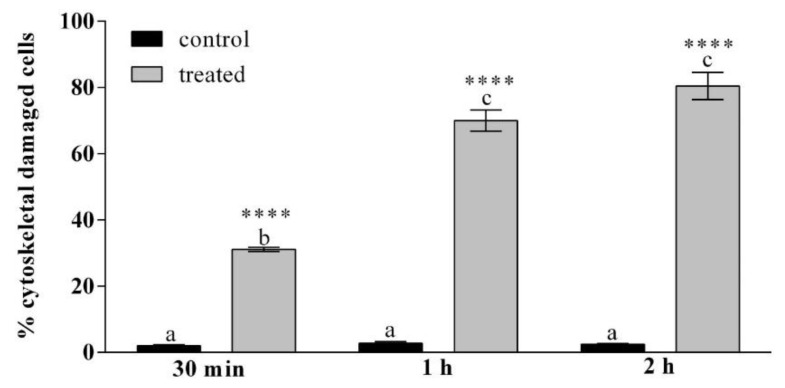
Percentage of haemocytes stained with phalloidin showing cytoskeletal damage, after treatment with OPs deriving from 2 equivalent females at different times of exposure (30 min, 1 h and 2 h). Data are presented as mean ± SD (*n* = 3). Statistical analysis was performed with one-way ANOVA and Bonferroni (for control vs. treatment analysis) and Tukey post-hoc test (for all sample analysis). Different letters indicate significant differences among all treatments (*p* value < 0.0001), asterisks indicate significant differences between control and treated samples at the same experimental time (*p* value < 0.0001).

In Figure 7 and in Table S4 the percentage of cells that show cytoskeletal damage on the total number of cells is reported. After 30 min of incubation with OPs the percentage of cells with cytoskeletal damage was equal to 31% of the total haemocytes, while no damage to actin filaments was observed in control cells. After 1 h the percentage rose to 70% for the treated cells. After 2 h 80% of the treated cells showed cytoskeletal damage.

### 3.3. In Vivo Encapsulation

**Figure 8:**
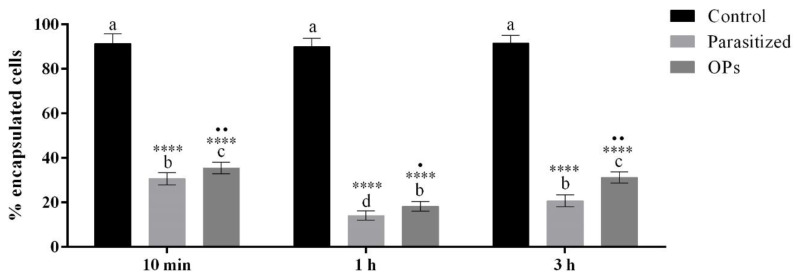
Percentage of encapsulation of chromatographic spheres extracted after 6 h from larvae injected with 1× PBS (control), after parasitization or OP treatment at 10 min, 1 h or 3 h before injection of spheres. Data are presented as mean ± SD (*n* = 3). Statistical analysis was performed with one-way ANOVA and Bonferroni (for control vs. treatment and parasitized vs. OPs analysis) and Tukey post-hoc test (for all sample analysis). Different letters indicate significant differences among all treatments (*p* value < 0.0001), asterisks indicate significant differences between control and treated samples at the same experimental time (*p* value < 0.0001), dots indicate significant differences between parasitized and OP treatment at the same experimental time (●● *p* value < 0.01, ● *p* value < 0.05).

To assess whether the alterations induced by OPs influenced the ability of the haemocytes to recognize and encapsulate foreign intruders, a test was performed by injecting OPs (2 females equivalent) or 1× PBS into *H. virescens* larvae at different times (10 min, 1 h and 3 h) before injection of chromatographic spheres used as non-self-material. Chromatographic spheres were injected also in *H. virescens* parasitized larvae. The spheres were classified according to the level of encapsulation as: 0 = unencapsulated (no haemocytes layer); 1 = the thickness of capsule is one or more than one layer, but less than a half of the bead’s radius; 2 = the thickness of capsule is equal or more than a half of the bead’s radius. We considered encapsulated exclusively the spheres of type 2 (Figure S1). Figure 8 and Table S5 show a strong reduction in the encapsulation capacity in the different treatments.

## 4. Discussion

Endoparasitoid insects have developed, during evolution, mechanisms capable to alter the physiology and biochemistry of the host, and they are able to create an environment suitable for the development of their progeny [4,27,39,52]. Such alterations imply the manipulation of the host’s immune system. The suppression of the immune reaction in hosts parasitized by endophagous hymenopterans is a very complex syndrome, characterized by several molecular mechanisms integrating each other [39,43,53]. The maternal factors, introduced into the host during the oviposition, are the main protagonists of the physiological alterations observed in the parasitized hosts [54,55]. These factors include venom proteins, symbiotic Polydnavirus (PDV), virus-like particles (VLP) and ovarian proteins (OPs) [4,15,16,49,52,56,57]. Among these, the PDVs have received a great interest over the years as an important source of useful genes [19]. PDVs are associated exclusively with endoparasitoid wasps of lepidopteran larvae [43,58]. PDVs replicate only in the cells of the wasp ovarian calyxes and are released in the lumen of the oviduct [28,29]. Once introduced into the host, PDVs infect tissues and actively express their genes [59]. The products of these genes induce a wide range of alterations to the neuroendocrine and immune system [27]. Many genes of viral origin have been isolated and characterized [4,24,26,38,44,45,60,61,62,63,64,65], but their expression requires several hours to be able to perform the immunosuppressive effect. Because the insect’s immune system is already functioning within minutes of recognizing the parasitoid, the parasitoid needs to alter its defense mechanisms in a short time [66]. Therefore, other maternal factors, such as OPs [28,49,67] and venom [15,56,68,69,70,71], or the proteins that cover and protect the parasitoid egg [72,73,74,75] must necessarily set to mediate immune-suppression in the early stages of parasitization. Luckhart and Webb [76] demonstrated the immunosuppressive intervention of OPs after 30 min from parasitization in *Campoletis sonorensis.* As already showed by Ferrarese et al. [39], there is a reduction of the haemocyte titer in the haemolymph following parasitization, probably due also to the action of the venom and/or OPs that induce, after few hours, a high cellular mortality. The molecular mechanisms that induce such alterations have not been clarified yet.

In this study we focused on the role of OPs in the host-parasitoid system *Heliothis virescens*-*Toxoneuron nigriceps.* OPs seem to interfere with the cell-mediated immune system in the minutes following parasitization, before *T. nigriceps* BracoVirus (*Tn*BV) infects host tissues and expresses its genes. The OPs induce a series of alterations affecting the haemocytes and carrying the cell to death.

In haemocytes treated with OPs many alterations are recorded, including mitochondrial alterations accompanied by ATP depletion, loss of transmembrane potential and the consequent increase in reactive oxygen species (ROS) in the cytoplasm. Mitochondria, therefore, can be a very important target in the attempt to alter cellular function. A similar picture has been described as one of the different manifestations of cell death [77]. Furthermore, several studies suggest that the alteration of the actin cytoskeleton is strongly linked to the increase in intracellular ROS levels and apoptosis [78,79]. ROS are physiologically present in cells at low concentrations and increase considerably in the case of severe mitochondrial damage [80,81]. In our system, haemocytes treated with OPs show that oxidative stress often occurs with cytoskeletal damage to actin filaments; these changes may explain the high levels of haemocyte mortality. The vacuolization observed in the haemocytes already after 30 min from exposure to OPs, confirms the observations reported by Ferrarese et al. [39] on haemocytes extracted from parasitized larvae. These high levels of oxidizing agents, together with a change in pH, probably linked to the increase of ROS, can negatively affect survival and cellular functionality [82,83]. All the observed alterations induce a loss of functionality in the haemocytes. In fact, by injecting OPs and chromatographic spheres into the larval haemocoel, a loss of encapsulation capacity by the haemocytes was observed. We observed a strong reduction of encapsulation both in parasitized larvae and when treated with OPs, as already reported for the endoparasitoid *Macrocentrus cingulum* in which OP treatment inhibited the encapsulation in a dose-dependent manner [84]. A *T. nigriceps* OP stronger effect was recorded at 1 h. This allowed the hypothesis in that the inhibition of haemocyte’s encapsulation capacity is caused not only by OPs but also by venom and *Tn*BV. Indeed, a previous study [15], among *T. nigriceps* venom proteins, identified the Calreticulin protein, whose role in inhibition of encapsulation has been reported in several parasitoids [85,86,87,88]. On the contrary in *M. cingulum* the mix of venom and OPs did not have a different effect compared to OPs alone, suggesting that, in this case, there are no synergic effect of venom and OPs in suppressing the encapsulation by *Ostrinia furnacalis* haemocytes [84]. Moreover, we observed a higher percentage of unencapsulated beads in larvae parasitized 3 h before injection of beads compared to larvae treated with OPs. This could be due to the expression of *Tn*BV genes that act on the host immune system [24,26].

The present work confirms the hypothesis that OPs have short-term immunosuppressive activity also in the host-parasitoid system *H. virescens*-*T. nigriceps*, as reported in other host-parasitoid system, such as *Chilo suppressalis*-*Cotesia chilonis* [89]. To guarantee the success of parasitization a synergic action of all parasitic factors is required. Several *Tn*BV genes have been reported to be responsible for morphological alterations and apoptosis in haemocytes, but their expression occurs at least after 3–4 h [24,26]. The fibrous layer on the *T. nigriceps* egg surface [47] venom proteins [15] and OPs ensure an escape from host cellular immune responses immediately after oviposition.

Knowledge of the sequence of biologically active proteins will probably allow us to understand the mechanisms by which they act.

## 5. Conclusions

Little information on the role of Ovarian Proteins (OPs) is available in the literature. Most of the studies concerning host-parasitoid systems, and in particular the parasitic factors, focus on Polydnavirus (PDV), if they are present, and venom. With this study we provide valuable information on possible functions of these secretions on host haemocytes, in the host-parasitoid system *Heliothis virescens*–*Toxoneuron nigriceps.* Experiments performed on haemocytes treated with OPs showed many alterations, including the increase of reactive oxygen species (ROS) in the cytoplasm and actin cytoskeleton disruption. These changes provoke both reduction in haemocyte functionality, losing encapsulation capacity, and increasing cellular death. Our study shows that OPs have a significant action, especially regarding the functional alteration of haemocytes, the cells of the host immune response. Overall OPs, together with other maternal factors (venom and PDV) actively contribute to suppress the host immune response, supporting the development of the parasitoid larva and the success of the parasitism.

This study underlines the importance of OPs and suggests further in-depth analysis to characterize the entire protein profile of this mixture of parasitic origin, and also specific analysis to identify the active proteins responsible for the described effects.

## Data Availability

Data is contained within this article and the supplementary material.

## Supplementary Materials

The following are available online at https://www.mdpi.com/2075-4450/12/1/33/s1, Protocol S1: Extraction and purification of *Toxoneuron nigriceps* Ovarian Proteins (OPs), Table S1: Data obtained counting haemocytes incubated with 1×PBS solution (control), haemocytes extracted from larvae 24 h after parasitization and OPs derived from 1 or 2 equivalent females, Table S2: Data obtained from the counts of healthy cells or treated with OPs at different times (30 min, 1 h and 2 h) showing vacuolization, Table S3: Data obtained from counting healthy cell or treated with OPs at different times (30 min, 1 h and 2 h) showing ROS positive cells, Table S4: Data obtained from haemocytes stained with phalloidin showing cytoskeletal damage, after OPs treatment at different exposure times (30 min, 1 h and 2 h), Table S5: Data obtained from haemocytes after injection of chromatographic spheres and after OPs treatment at different exposure times (30 min, 1 h and 2 h) or parasitization, Figure S1: Chromatographic spheres extracted from 5 larvae for each time (10 min (a), 3 h (b) and 6 h (c) after OP injection, Figure S2: Healthy haemocytes or treated with OPs for 2 h and stained with DAPI and H_2_DCFDA.
