# Supplementary material for: Role of Ovarian Proteins Secreted by Toxoneuron nigriceps (Viereck) (Hymenoptera, Braconidae) in the Early Suppression of Host Immune Response
Source: Insects. 2021 Jan 5;12(1):33. doi: 10.3390/insects12010033 (PMC7824821; doi:10.3390/insects12010033)
Supplement: Supplementary file 1 [file insects-12-00033-s001.zip › supplementary-xml/Figure S1.pdf]

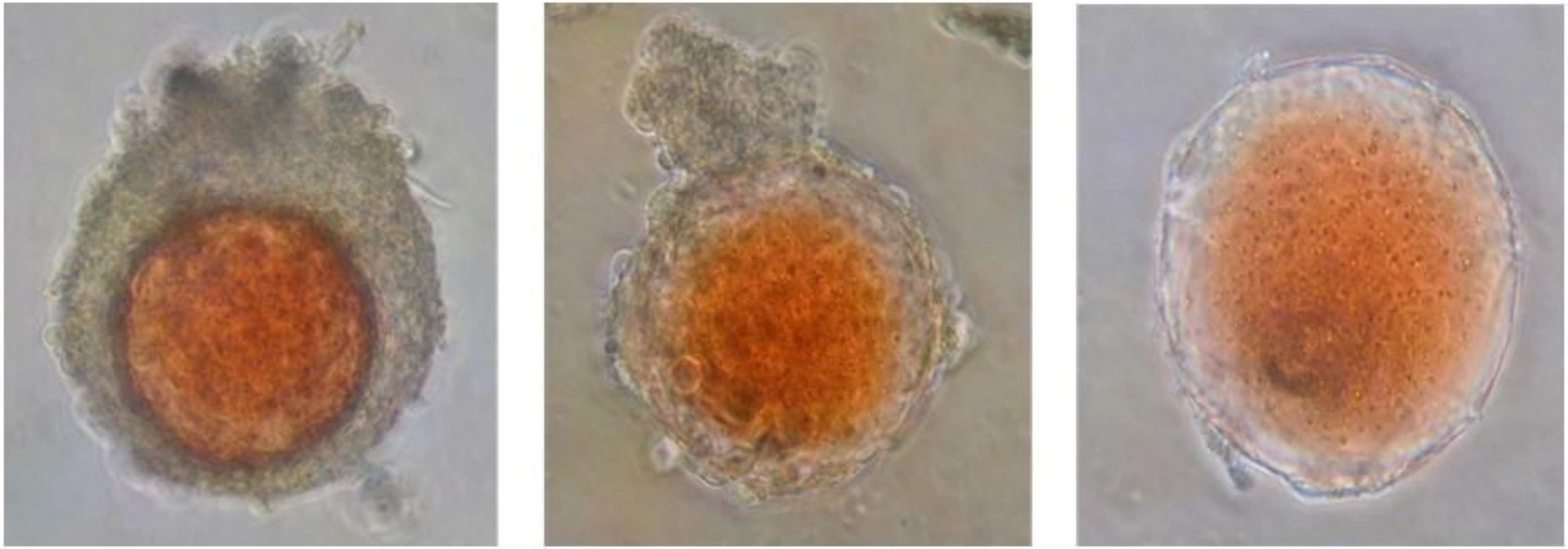

a b c

Figure S1. Chromatographic spheres extracted from 5 larvae for each time (10 min (**a**), 3 h (**b**) and 6 h (**c**) after OP injection, (n = 3 replicates). We classified the following level of encapsulation: 0, unencapsulated (no haemocytes layer); 1, the thickness of capsule is one or more than one layer, but less than a half of the bead's radius; 2, the thickness of capsule is equal or more than a half of the bead's radius.
