# Supplementary material for: Role of Ovarian Proteins Secreted by Toxoneuron nigriceps (Viereck) (Hymenoptera, Braconidae) in the Early Suppression of Host Immune Response
Source: Insects. 2021 Jan 5;12(1):33. doi: 10.3390/insects12010033 (PMC7824821; doi:10.3390/insects12010033)
Supplement: Supplementary file 1 [file insects-12-00033-s001.zip › supplementary-xml/Figure S2.pdf]

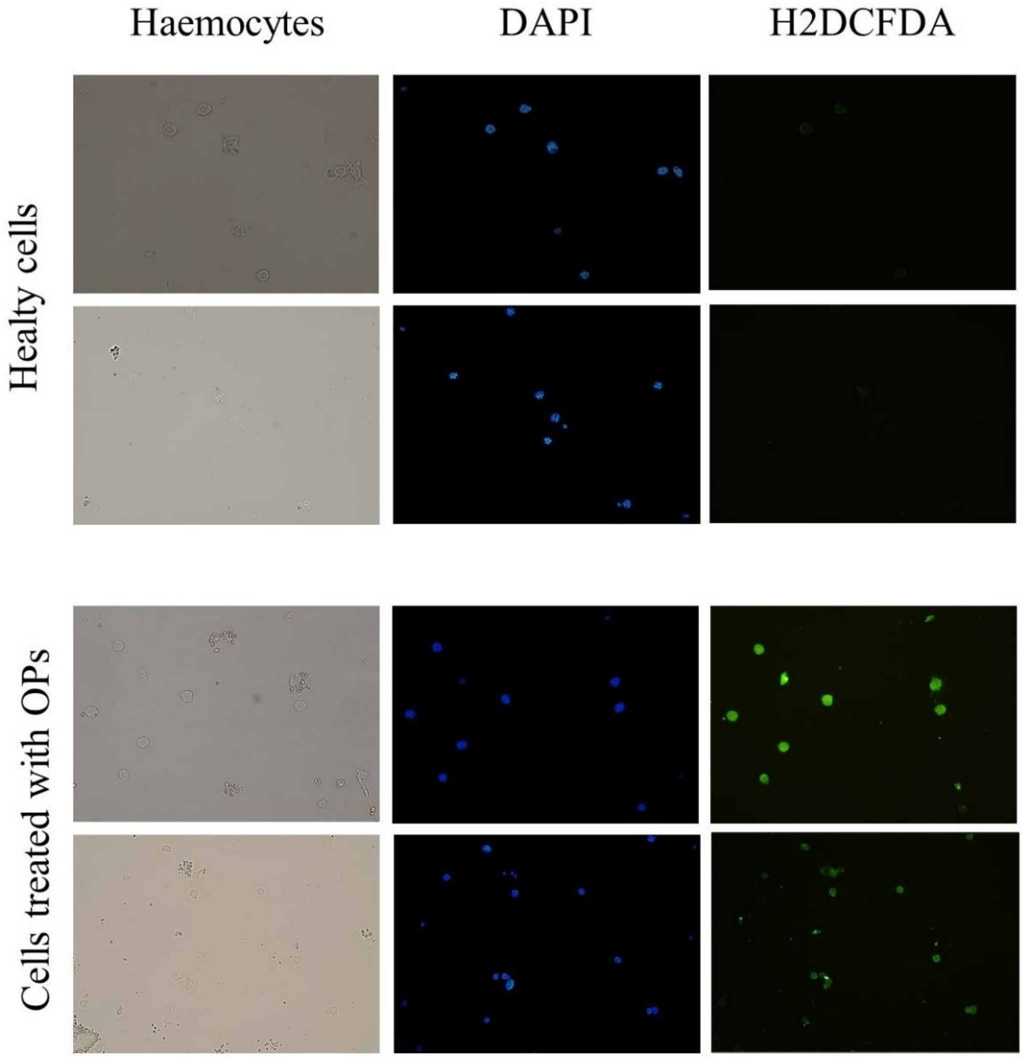

Figure S2. The haemocytes incubated with OPs (deriving from 2 equivalent females) at 2 h or with 1× PBS (negative control) were detached from the well transferred on slides and subjected to different staining methodologies. For H_2DCFDA staining, after fixed on slides, cells were incubated in the dark with H_2DCFDA 10 μM for 30 min at room temperature. After three times washing in 1× PBS, the slides were mounted with Fluoroshield with DAPI, histology mounting medium (Sigma-Aldrich St Louis, MO, USA, catalogue number F6057).
