# Supplementary material for: Role of Ovarian Proteins Secreted by Toxoneuron nigriceps (Viereck) (Hymenoptera, Braconidae) in the Early Suppression of Host Immune Response
Source: Insects. 2021 Jan 5;12(1):33. doi: 10.3390/insects12010033 (PMC7824821; doi:10.3390/insects12010033)
Supplement: Supplementary file 1 [file insects-12-00033-s001.zip › supplementary-xml/Protocol S1.pdf]

Extraction and purification of *Toxoneuron nigriceps* ovarian proteins (OPs)

OPs are one of the components of the *T. nigriceps* ovarian calix fluid, together with Polydnavirus (PDV) particles. The ovarian fluid starts to be produced by ovarian calix cells during the pupal stage of *T. nigriceps* females and it is then continuously produced during the entire life cycle by the adult female. During the oviposition, the ovarian calix fluid is injected along with the venom in larval stages of the host contributing to the parasitization success.
Below we report the detailed protocol of purification of ovarian proteins from the fluid of the ovarian calyx and of the separation from the PDV particles.

• Prepare a Petri dish with drops of 1× PBS solution (20 μL for one or two females)

• Anesthetize females with carbon dioxide or putting them on ice

• Place the insect with its abdomen up and remove the ovipositor to which the ovaries are linked, pulling it away with a pair of forceps

• Put the ovaries in 1× PBS solution (20 μL for ovaries from one or two females)

• After the ovary collection, dissect them to the point of the translucent part

• Aspirate the liquid with a Gilson and transfer it to a 1.5 mL Eppendorf tube

• Centrifuge at 2,000 g for 5 min. at 4 ° C, to remove the eggs and the calyx tissues and any other debris

• Transfer the supernatant to a new 1.5 mL Eppendorf tube

• Filter the supernatant with a 0.45 μm Millex PVDF filters

• Wash 3 times with 1 mL of 1× PBS

• Centrifuge at 30,000 g for 1 hour at 4 ° C to separate the *Tn*BV particles

• Transfer the supernatant, containing OPs, into Millipore cut-off 3000 tubes

• Centrifuge at 3,000 g until you have a volume of about 500 μL

• Store OPs at –80 ° C until the use.
