# Supplementary material for: Role of Ovarian Proteins Secreted by Toxoneuron nigriceps (Viereck) (Hymenoptera, Braconidae) in the Early Suppression of Host Immune Response
Source: Insects. 2021 Jan 5;12(1):33. doi: 10.3390/insects12010033 (PMC7824821; doi:10.3390/insects12010033)
Supplement: Supplementary file 1 [file insects-12-00033-s001.zip › supplementary-xml/Table S1.pdf]

Table S1. Data obtained counting haemocytes incubated with Pringle solution (control), haemocytes extracted from larvae 24 h after parasitization and OPs derived from 1 or 2 equivalent females. Data are presented as mean ± SD (n = 3). Statistical analysis was performed with one-way ANOVA (analysis of variance) and Bonferroni *post-hoc* test. Different letters indicate significant differences (p value < 0.0001).

	**Control**	**Parasitized**	**OPs 1 Female Eq.**	**OPs 2 Female Eq.**
**Total cell number**	132.67 ± 3.06	156 ± 13.23	153 ± 14.42	142 ±1.73
**Number of viable cells**	112.67 ± 6.51	46 ± 4	52.67 ± 1.15	54 ± 1
**% of cellular vitality**	84.87 ± 3.05a	29.59 ± 3.19b	34.58 ± 2.48bc	38.04 ± 1.12c
