# Supplementary material for: Role of Ovarian Proteins Secreted by Toxoneuron nigriceps (Viereck) (Hymenoptera, Braconidae) in the Early Suppression of Host Immune Response
Source: Insects. 2021 Jan 5;12(1):33. doi: 10.3390/insects12010033 (PMC7824821; doi:10.3390/insects12010033)
Supplement: Supplementary file 1 [file insects-12-00033-s001.zip › supplementary-xml/Table S2.pdf]

Table S2. Data obtained from the counts of healthy cells or treated with OPs at different times (30 min, 1 h and 2 h) showing vacuolization. Data are presented as mean ± SD (n = 3). Different letters indicate significant differences among all treatments (p value < 0.0001), asterisks indicate significant differences between control and treated samples at the same experimental time (** p value < 0.01; **** p value < 0.0001).

	**30 min**		**1 h**		**2 h**	
	**Control**	**Treated**	**Control**	**Treated**	**Control**	**Treated**
**Total cell number**	161.33 ± 13.32	142 ± 4	144.33 ± 6.51	135 ± 6.24	138 ± 6.08	131.67 ± 9.02
**Number of vacuolated cells**	24 ± 4.58	37.67 ± 7.37	22.67 ± 1.53	46.67 ± 4.73	21.67 ± 2.52	54 ± 1
**% of vacuolated cells**	14.80 ± 1.85a	26.56 ± 5.34b **	15.71 ± 0.83a	34.71 ± 4.92bc ****	15.68 ± 1.30a	41.16 ± 3.27c ****
