# Supplementary material for: Role of Ovarian Proteins Secreted by Toxoneuron nigriceps (Viereck) (Hymenoptera, Braconidae) in the Early Suppression of Host Immune Response
Source: Insects. 2021 Jan 5;12(1):33. doi: 10.3390/insects12010033 (PMC7824821; doi:10.3390/insects12010033)
Supplement: Supplementary file 1 [file insects-12-00033-s001.zip › supplementary-xml/Table S3.pdf]

Table S3. Data obtained from counting healthy cell or treated with OPs at different times (30 min, 1 h and 2 h) showing ROS positive cells. Data are presented as mean ± SD ($n = 3$). Different letters indicate significant differences among all treatments (p value < 0.0001), asterisks indicate significant differences between control and treated samples at the same experimental time (p value < 0.0001).

	30 min		1 h		2 h	
	**Control**	**Treated**	**Control**	**Treated**	**Control**	**Treated**
**Total cell number**	137.67 ± 9.24	136.67 ± 4.04	135.67 ± 2.89	131.33 ± 2.08	140.67 ± 2.08	129 ± 7.21
**Number of cells ROS positive**	1.67 ± 0.58	63 ± 3.46	2.67 ± 0.58	84.67 ± 1.53	2.33 ± 1.53	116 ± 6.08
**% of cells showing oxidative stress**	1.22 ± 0.46a	46.10 ± 2.16b ****	1.96 ± 0.41a	64.48 ± 1.51c ****	1.67 ± 1.10a	89.95 ± 1.85d ****
