# Supplementary material for: Role of Ovarian Proteins Secreted by Toxoneuron nigriceps (Viereck) (Hymenoptera, Braconidae) in the Early Suppression of Host Immune Response
Source: Insects. 2021 Jan 5;12(1):33. doi: 10.3390/insects12010033 (PMC7824821; doi:10.3390/insects12010033)
Supplement: Supplementary file 1 [file insects-12-00033-s001.zip › supplementary-xml/Table S4.pdf]

Table S4. Data obtained from haemocytes stained with phalloidin showing cytoskeletal damage. after OPs treatment at different exposure times (30 min. 1 h and 2 h). Data are presented as mean ± SD ($n = 3$). Data are presented as mean ± SD ($n = 3$). Different letters indicate significant differences among all treatments (p value < 0.0001). asterisks indicate significant differences between control and treated samples at the same experimental time (p value < 0.0001).

	30 min		1 h		2 h	
	**Control**	**Treated**	**Control**	**Treated**	**Control**	**Treated**
**Total cell number**	131 ± 3.61	133 ± 1.73	135 ± 2.65	132 ± 2.65	137 ± 1.73	130 ± 4.58
**Number of cells showing cytoskeletal damage**	2.67 ± 0.58	41.33 ± 1.15	3.67 ± 1.15	92.33 ± 6.81	3.33 ± 0.58	104.67 ± 11.59
**% of cells showing cytoskeletal damage**	2.04 ± 0.46a	31.09 ± 1.13b ****	2.72 ± 0.90a	69.98 ± 5.51c ****	2.43 ± 0.41a	80.44 ± 7.09c ****
