# Supplementary material for: Role of Ovarian Proteins Secreted by Toxoneuron nigriceps (Viereck) (Hymenoptera, Braconidae) in the Early Suppression of Host Immune Response
Source: Insects. 2021 Jan 5;12(1):33. doi: 10.3390/insects12010033 (PMC7824821; doi:10.3390/insects12010033)
Supplement: Supplementary file 1 [file insects-12-00033-s001.zip › supplementary-xml/Table S5.pdf]

Table S5. Data obtained from haemocytes after injection of chromatographic spheres and after OPs treatment at different exposure times (30 min, 1 h and 2 h) or parasitization. Data are presented as mean ± SD ($n = 3$). Different letters indicate significant differences among all treatments, asterisks indicate significant differences between control and treated samples at the same experimental time, dots indicate significant differences between parasitized and OP treatment at the same experimental time (p value = 0.005).

	10 min			1 h			3 h		
	**Control**	**Parasitized**	**OPs**	**Control**	**Parasitized**	**OPs**	**Control**	**Parasitized**	**OPs**
**% of encapsulated cells**	91.40 ± 4.35a	26.70 ± 1.79b ****	35.50 ± 1.60c ****/••	90.02 ± 3.75a	12.10 ± 1.08d ***	20.28 ± 0.82b ****/•	91.57 ± 3.49a	20.75 ± 2.72b ****	31.27 ± 2.52c ****/••
